# Comparison of Lower and Upper Quarter Y Balance Test Performance in Adolescent Students with Borderline Intellectual Functioning Compared to Age- and Sex-Matched Controls

**DOI:** 10.3390/children8090805

**Published:** 2021-09-14

**Authors:** Julian Bauer, Helena Kammermeier, Gerrit Schwiertz, Thomas Muehlbauer

**Affiliations:** Division of Movement and Training Sciences/Biomechanics of Sport, University of Duisburg-Essen, 45141 Essen, Germany; helena.kammermeier@stud.uni-due.de (H.K.); gerrit.schwiertz@uni-due.de (G.S.); thomas.muehlbauer@uni-due.de (T.M.)

**Keywords:** postural control, shoulder mobility/stability, mental deficits, atypical development

## Abstract

The Lower (YBT-LQ) and Upper (YBT-UQ) Quarter Y Balance Test are well established assessment tools for the examination of dynamic balance and shoulder mobility/stability, respectively. However, investigations on YBT-LQ/UQ performance in students with borderline intellectual functioning (BIF) (i.e., intelligence quotient of 70–84 etc.) are lacking. Thus, the aim of the study was to compare YBT-LQ/UQ performance in students with and without BIF. Thirty students with BIF (age: 13.7 ± 1.2 years) and 30 age-/sex-matched students without BIF (age: 13.7 ± 1.3 years) performed the YBT-LQ and/or YBT-UQ. Normalized maximal reach distances (% leg/arm length) per reach direction and the composite score were used as outcome measures. A univariate analysis of variance was conducted to test for significant group differences. Irrespective of limb and reach direction, students with BIF compared to those without BIF showed significantly worse YBT-LQ (*p* ≤ 0.001–0.031; Cohen’s *d* = 0.57–1.26) and YBT-UQ (*p* ≤ 0.001–0.015; Cohen’s *d* = 0.68–1.52) performance with moderate to large effect sizes. Due to the poorer performance levels of students with BIF, specifically tailored interventions should be developed that have the potential to improve their dynamic balance and shoulder mobility/stability.

## 1. Introduction

Borderline intellectual functioning (BIF) is a condition in a group of people who are on the threshold between normal intellectual functioning and intellectual disability [1]. The Diagnostic and Statistical Manual of Mental Disorders defines the corresponding intelligence quotient (IQ) in the 70–84 range “between 1 and 2 standard deviations below the mean on the normal curve of the distribution of intelligence” [2]. BIF is not a single condition limited to a single neurodevelopmental syndrome, but rather a meta-condition associated with a variety of cognitive difficulties [3]. Peltopuro et al. [4] reported 13.6% of the world-wide population to fit into the BIF category. It is described as a potential cost-intensive condition due to possible tuition, medications, and remedial education needs [5]. 

A proposed etiology of BIF is an atypical functioning of the brain [6]. Baglio et al. [3] demonstrated that abnormal gray matter (GM) development correlated with decreased IQ levels in children with BIF. Therefore, abnormal cortical and subcortical GM development, and increased GM volume in bilateral sensorimotor and right posterior temporal cortices [3] accompanied with possible co-factors such as a chaotic upbringing, inadequate parenting as well as a low IQ of the parents [7] may be responsible for the presence of BIF.

A link between motor skills and cognitive development was already proposed by Piaget and Inhelder [8]. This association between motor and cognitive development may be explained by the co-activation of the cerebellum, being responsible for complex and coordinated movements and the prefrontal cortex, being responsible for higher order executive functioning [9]. One substantial motor skill is balance which is related both to sport-specific skills as well as activities of daily living.

As BIF is mainly associated with impairments in cognitive abilities, only few studies [10,11] have assessed motor abilities of students with BIF. However, no study assessed dynamic balance and shoulder mobility/stability which are important pre-requisites to reach motor competence/skills, enabling participation in sports and physical activities. Therefore, the rationale of the study was to gain knowledge on the topic, so that in case of differences between BIF and non-BIF students, physical interventions can be implemented. Such knowledge is additionally important because performance differences of the lower and upper quarter are of relevance for sports participation at school or sport clubs and for activities of daily living as well.

In this regard, Kaupuzs and Larins [10] performed the modified Clinical Test of Sensory Interaction on Balance which assesses static balance in four different testing conditions. The students with BIF at the age of 11–13 years displayed significantly worse results when standing with (1) eyes open on a firm surface, (2) eyes closed on a firm surface, and (3) eyes open on a foam surface when compared with typically developed students, while there were no significant differences when standing with (4) eyes closed on a foam surface. In addition, Alesi et al. [11] used the Test of Gross Motor Development (TGMD-2) and compared children with Down syndrome, with BIF, and typically developed children. The authors revealed significant differences in overall gross motor skills, locomotion, and object control skills between all groups, with the BIF group achieving better results than the Down syndrome group but worse results than the typically developed group in all three categories.

A small number of studies compared the motor proficiency of students with versus without BIF showing that the former performed worse than the latter. Therefore, the aim of the present study was to compare dynamic balance and shoulder mobility/stability of students with versus without BIF. With reference to the reviewed literature [10,11] we hypothesized that students with BIF would display worse performance levels in dynamic balance as assessed through the Lower Quarter Y Balance Test (YBT-LQ) and in shoulder mobility/stability as assessed through the Upper Quarter Y Balance Test (YBT-UQ) compared to students without BIF.

## 2. Materials and Methods

### 2.1. Participants

The participants were recruited from different schools for students with special needs in the field of learning and/or socio-emotional deficits. The following selection criteria were applied. People were included if they were previously assessed by psychologists and special school teachers and diagnosed with an IQ of 70–84, therefore being classified as having BIF. People were excluded from study participation if they (1) were outside of the aforementioned age category, (2) had a musculoskeletal or neurological disorder during the last three months prior to the beginning of the study, (3) had other medical conditions that could have affected their ability to execute the YBT-LQ/UQ. As all of the assessed students attend a school for students with special needs, the requirement of the more recent suggestion of the American Psychiatric Association [12] describing the condition as being the focus of clinical attention or having an impact on the individual’s treatment and prognosis, was therefore fulfilled. The reference data were obtained from sex- and age-matched controls who were assessed during several testing occasions with the same testing protocol. As not all, but most of the controls performed both the YBT-LQ/UQ, thus the control group mostly overlapped but was not exactly the same. The students without BIF were recruited from randomly chosen urban public schools in the Ruhr metropolitan area.

None of the subjects had prior experience in the execution of the YBT-LQ/UQ. The heads of the schools and the class teachers were informed about the study’s objective as well as the testing procedure and gave their written consent. In addition, parents’ written consent and the participants’ assent was obtained prior to the beginning of the study. The study was carried out according to the Declaration of Helsinki and the study protocol was approved by the Human Ethics Committee at the University of Duisburg-Essen, Faculty of Social Sciences (TM_23.03.2020, approval date is 23 March 2020)

The characteristics of the study participants by student group are shown in Table 1. We did not observe statistically significant differences between the groups.

### 2.2. Measurements

Testings were carried out on two different measurement days for each school. The testings were conducted at the same time in the morning in a room prepared for the assessments. Testing personnel consisted of trained professionals who were experienced raters in the YBT-LQ/UQ. A standardized verbal instruction was given prior to each test.

### 2.3. Anthropometry

Body mass was assessed fully dressed in normal casual clothes without shoes to the nearest 100 g with an electronical scale (Seca 803, Basel, Switzerland). Body height was assessed with a stadiometer (Seca 217, Basel, Switzerland), also without shoes, to the nearest 0.5 cm. Left and right arm length (AL) was measured with a cloth tape attached to the wall from the seventh cervical spinous process to the distal tip of the middle finger with the shoulder abducted to 90° [13]. Left and right leg length (LL) was measured from the anterior iliac spine to the most distal part of the medial malleolus using cloth tape with the participants lying supine [14].

### 2.4. Y Balance Tests–General Execution Details

Both the YBT-LQ and YBT-UQ were executed with a commercial YBT test kit (Functional Movement Systems^®^, Chatham, PA, USA). Standardized instructions were given before the execution and a demonstration trial was performed by a member of the testing personnel. The movement was demonstrated in a manner that was understood by every participant which was assured by a verbal confirmation by the participants.

Three practice trials were performed followed by three data collection trials for both the YBT-LQ and YBT-UQ, respectively. A rest of one minute between each trial was granted. The best trial (i.e., maximal reach distance in cm) for each leg (YBT-LQ) and arm (YBT-UQ) and reach direction was used for further analysis.

### 2.5. Lower Quarter Y Balance Test

**Figure 1:**
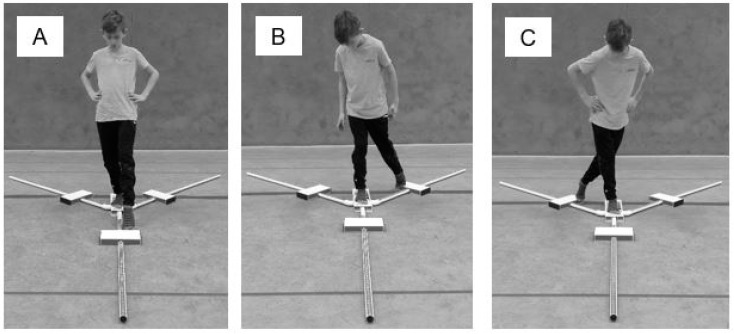
Setup for the assessment of Lower Quarter Y Balance Test performance with (**A**) anterior reach, (**B**) posteromedial reach, and (**C**) posterolateral reach. Upper Quarter Y Balance Test.

The participants were asked to move the mobile reach indicator as far as possible in the anterior (AT) direction with the left leg while standing on the central platform on the right leg. The same protocol was performed for the posteromedial (PM) and the posterolateral (PL) direction afterwards (see Figure 1). 

**Figure 2:**
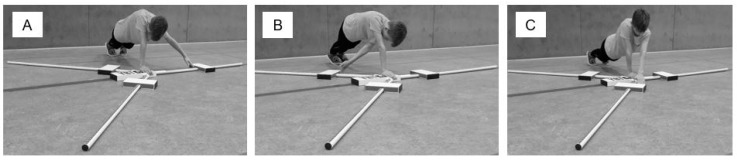
Setup for the assessment of Upper Quarter Y Balance Test performance with (**A**) medial reach, (**B**) inferolateral reach, and (**C**) superolateral reach.

Participants were instructed to move the mobile reach indicators as far as possible in the medial (MD) direction starting with the left arm as the mobile arm, while maintaining a one-arm push-up position with the right arm on the central platform. Immediately after reaching in the MD direction, the participants had to move the reach indicator to the inferolateral (IL) and superolateral direction (SL) while maintaining the one-arm push-up contact to the platform (see Figure 2). Trials were classified as invalid if the subjects lost three-point contact (i.e., both legs and the immobile arm shoulder-width on the floor, touched the floor with the mobile arm or actively pushed the reach indicator) [15]. 

### 2.6. Data and Statistical Analyses

Normalized maximal reach distances (% AL/LL) per reach direction and arm/leg were calculated by dividing the absolute maximal reach distance (cm) by LL for the YBT-LQ and by AL for the YBT-UQ and then multiplied by 100. The normalized (% AL/LL) composite score (CS) was computed for each leg and arm as the sum of the maximal reach distance (cm) per reach direction and then divided by three times LL for the YBT-LQ/UQ and three times AL for the YBT-UQ and then multiplied by 100 [16].

Descriptive data of the dependent variables are presented as group mean values and standard deviations. A univariate analysis of variance was conducted to test for significant differences between students with versus without BIF. The significance level was set at *p* < 0.05. Further, Cohen’s *d* was used as an effect size measure and classified as representing small (0 ≤ *d* ≤ 0.49), moderate (0.50 ≤ *d* ≤ 0.79), and large effects (*d* ≥ 0.80). All statistical analyses were conducted using Statistical Package for Social Sciences, Version 27.0 (SPSS Inc., Chicago, IL, USA).

## 3. Results

**Figure 3:**
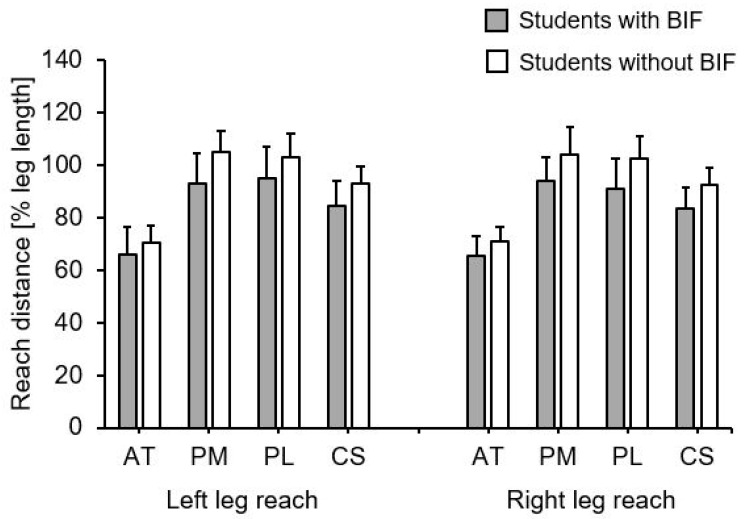
Group mean values and standard deviations for the normalized Lower Quarter Y Balance Test performance by student group. Notes: AT = anterior reach; BIF = borderline intellectual functioning; CS = composite score; PL = posterolateral reach; PM = posteromedial reach.

Group mean values and standard deviations for the normalized YBT-LQ performance by student group are presented in Table 2 and Figure 3. Irrespective of outcome measure, we found significantly lower values in students with compared to without BIF. The respective effect sizes ranged between moderate to large.

**Figure 4:**
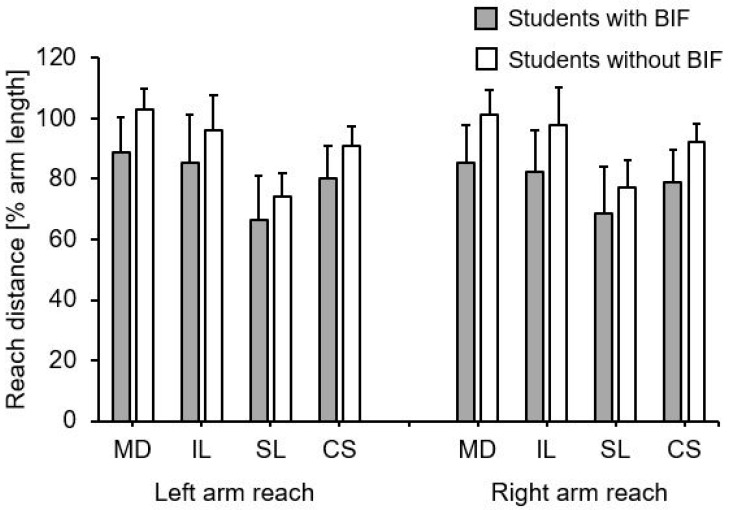
Group mean values and standard deviations for the normalized Upper Quarter Y Balance Test performance by student group. Notes: BIF = borderline intellectual functioning; CS = composite score; IL = inferolateral reach; MD = medial reach; SL = superolateral reach.

Table 3 and Figure 4 display group mean values and standard deviations for the normalized YBT-UQ performance by student group. Again, we observed significantly smaller values for the students with compared to those without BIF. The corresponding effect sizes ranged from moderate to large.

## 4. Discussion

To the best of our knowledge, the present study is the first to compare dynamic balance and shoulder mobility/stability between students with and without BIF. Our main result was that students with BIF displayed significantly worse YBT-LQ and YBT-UQ performances compared to age-/sex-matched students without BIF. Respective effect sizes were moderate to large. These findings are in line with our initial hypotheses as well as with findings from Hartman et al. [17] and Westendorp et al. [18] who assessed gross motor skills based on the TGMD-2 in children with BIF and mild intellectual disabilities comparing them with their typically developed peers. In both studies, children with BIF scored lower in the subtests of the TGMD-2 compared to the typically developed children. In terms of balance, Vujik et al. [6] reported that children with BIF and children with mild ID showed significantly lower static balance, dynamic balance while moving fast, and dynamic balance while moving slowly compared to an age-/sex-matched normative population.

What might be the reasons for impaired YBT-LQ/UQ performances in students with BIF compared to age-/sex-matched students without BIF? First, the association between motor skills and cognitive development dates back to Piaget and Inhelder [8]. A relationship in terms of degrees of ID and deficits in motor proficiency was confirmed by Jeoung [19] who reported the mean rate of mastery of motor proficiency based on the Bruininks–Oseretsky Test was lowest in children with moderate disability (38.96%), followed by those with developmental disability (47.4%), those with autism (58.65%), those with mild ID (58.78%), and those with BIF (68.8%). The association between motor and cognitive development may be explained by the co-activation of the cerebellum, being responsible for complex and coordinated movements and the prefrontal cortex, being responsible for higher order executive functioning [9]. Second, the YBT-LQ/UQ require rather complex motor skills. On a mechanical level, the absence of normal proximal postural tension may lead to an insufficient stabilization of the trunk and the shoulder when executing a postural mobility and stability test like the YBT-LQ/UQ. This may be especially true for the YBT-LQ which is executed in an upright position which may lead to excessive forward and backward body tilting or lateral displacement [20]. In addition, divided attention is needed. More precisely, the participants were asked to combine a balance component, i.e., supporting the body with the immobile leg (YBT-LQ) or arm (YBT-UQ) and executing a goal-/object-directed component when moving the reach indicator with the mobile leg or arm. This combination of challenges, i.e., divided attention or dual-tasking may be impaired in students with ID. In this regard, children with Down syndrome showed significantly greater postural sway during sit-to-stand phases when concurrent motor tasks, such as holding a plastic cup, as part of a dual-task execution, had to be performed [21]. Third, the physical activity levels of students with BIF are reported to be lower than those of their typically developed peers [18]. Therefore, motor proficiency in terms of dynamic balance and shoulder mobility/stability may be impaired in students with BIF. Fourth, students with BIF are less represented in institutionalized sport clubs and physical activities [18]. This may be due to a limited precision of movements, feeling of uncertainty of posture and the fear of falling which may additionally strengthen their sedentary lifestyle [22]. Sedentariness is also reported to be a possible reason for multimorbidity throughout the lifespan of people with ID [23] who are frequently more often overweight and obese [24]. This lower sports and physical activity participation may additionally have a detrimental effect on the required goal-directed movement of the YBT-LQ/UQ (e.g., moving the reach indicator) and the accompanied balance demands. Fifth, balance relies on sensory systems (visual, proprioceptive and vestibular) and their mutual integration [25]. An insufficient level of maturity of these sensory systems may therefore impair motor function of BIF students [26].

Based on the results of two reviews, motor development of young people with ID can be improved by means of physical activity interventions [26,27]. Therefore, specific interventions should be developed to increase physical activity and facilitate motor development in students with BIF. The definite need of such interventions is highlighted by the fact that in the present study students with BIF scored lower in every tested age category compared to the YBT-LQ/UQ reference values of Schwiertz et al. [28,29,30] who assessed age-/sex-matched persons without BIF. The lower performance levels of students with BIF seem to be a stable finding across adolescence. Further, to be able to differentiate between low and high performers, YBT-LQ/UQ reference values should be established for students with BIF. Lastly, interventions focusing on balance and shoulder mobility/stability in children with BIF should be implemented early in order to decrease or even neutralize the detrimental effects of their impairment. As there is a lack of studies on interventions for people with BIF, only studies on people with ID can be considered as a reference. For example, interventions using Tai chi exercises [31] and balance/strength exercises [32,33] in young people with mild ID were effective to improve different parameters of balance and/or functional mobility/strength. These interventions together with Wii Fit balance game training [34] and rope-skipping exercises [35] demonstrated the largest effects (Hedges g > 0.80) on balance and strength. In addition, Lee et al. [36] reported a 40 minutes per day, two times a week, eight week-long balance training to be effective in improving the one-legged stance, the timed up-and-go, the 10-m walk, and the sit to stand test in a 14–19 year-olds training group with ID compared to a control group. Based on the proposed reasons for these worse results in students with BIF, the following implications arise: 1. Motor tasks with divided attention should be integrated into the training programs of young people with BIF, as these integrate both cognitive and motor tasks. 2. The physical activity levels of students with BIF need to be increased as sports participation may slow down or even neutralize the possible detrimental effects of BIF. 3. A variety of different stimuli on the visual, proprioceptive, and vestibular level should be integrated within these interventions as this variety proved to be highly effective for the development of the sensorimotor system.

### Limitations

Several limitations of this study need to be addressed. In people with ID such as the more severe forms of intellectual functioning deficit compared to BIF, Hove [37], Lahtinen et al. [24] and Lipowicz et al. [25] reported that the higher the IQ in people with ID, the better the balance and/or motor performance. However, it remains unclear whether this reported relationship is also present in dynamic balance and shoulder mobility/stability as assessed through the YBT-LQ/UQ in students with BIF. Therefore, differentiating the BIF group in terms of the severity of their deficits (i.e., being closer to typically developed students in case of higher IQs or being closer to students with ID in case of IQs on the lower end of the scale) might have confirmed this relationship. Second, a possible heterogeneity in terms of socio-emotional or other accompanying deficits may have altered the present results. Third, as the present results are only based on two schools of students with BIF, the findings cannot be generalized to other age groups with BIF. Fourth, the prevalence of BIF in boys and girls may be unequally distributed and also display different degrees of severity. Therefore, differentiating boys and girls in larger samples might have revealed additional sex-specific differences, as for example male adolescents have been reported to display worse results than female adolescents with BIF in terms of static balance performance [10].

## 5. Conclusions

Our study investigated differences in dynamic balance and shoulder mobility/stability between students with and without BIF. The results indicate that students with BIF display significantly lower performances compared to age- and sex-matched students without BIF. Our results point at the necessity to develop specific programs and to increase sport participation of young people with BIF to target the reported deficiencies in this population. Therefore, school settings may be very important in establishing physical activity routines in the lives of young people. Future research should assess whether the positive influence of interventions which were achieved in people with ID also prove to be effective for young people with BIF and whether the deficiencies in young people with BIF also exist in older people with BIF.

## Figures and Tables

**Table 1:** Characteristics of the study participants by student group.

Characteristic	30 Students with BIF (YBT-LQ/UQ)	30 Students without BIF (YBT-LQ)	30 Students without BIF (YBT-UQ)	*p*-Value (YBT-LQ)	*p*-Value (YBT-UQ)
Age (years)	13.7 ± 1.2	13.7 ± 1.3	13.7 ± 1.2	0.84	1.00
Sex (m/f)	22/8	22/8	22/8		
Body mass (kg)	58.6 ± 11.5	56.1 ± 11.0	53.4 ± 11.6	0.39	0.09
Body height (cm)	163.0 ± 7.9	166.1 ± 10.6	167.0 ± 11.1	0.21	0.11
Arm length, left (cm)	81.5 ± 4.7	–	83.0 ± 6.6	–	0.34
Arm length, right (cm)	81.0 ± 4.8	–	83.5 ± 7.1	–	0.12
Leg length, left (cm)	90.0 ± 5.2	91.3 ± 6.9	–	0.41	–
Leg length, right (cm)	90.0 ± 5.3	91.2 ± 6.3	–	0.42	–

Data are mean values ± standard deviations. BIF = borderline intellectual functioning; f = female; m = male; YBT-LQ = Lower Quarter Y Balance Test; YBT-UQ = Upper Quarter Y Balance Test.

**Table 2:** Lower Quarter Y Balance Test performance for students with versus without BIF.

Measure	Students with BIF (*n* = 30)	Students without BIF (*n* = 30)	*p*-Value	*d*-Value
Right leg reach				
AT (% LL)	65.3 ± 7.6	70.8 ± 5.6	0.002	0.82
PM (% LL)	94.1 ± 9.1	104.3 ± 10.2	<0.001	1.05
PL (% LL)	91.2 ± 11.2	102.8 ± 8.1	<0.001	1.19
CS (% LL)	83.5 ± 7.9	92.6 ± 6.5	<0.001	1.26
Left leg reach				
AT (% LL)	65.9 ± 10.4	70.7 ± 6.1	0.031	0.57
PM (% LL)	93.3 ± 11.3	105.1 ± 8.0	<0.001	1.23
PL (% LL)	95.1 ± 11.8	103.3 ± 8.8	0.003	0.77
CS (% LL)	84.7 ± 9.6	93.1 ± 6.4	<0.001	1.02

Data are mean values ± standard deviations. Cohen’s d with 0 ≤ d ≤ 0.49 indicating small, with 0.50 ≤ d ≤ 0.79 indicating moderate, and with d ≥ 0.80 indicating large effects. AT = anterior; BIF = borderline intellectual functioning; CS = composite score; LL = leg length; PL = posterolateral; PM = posteromedial [13].

**Table 3:** Upper Quarter Y Balance Test performance for students with versus without BIF.

Variables	Students with BIF (*n* = 30)	Students without BIF (*n* = 30)	*p*-Value	*d*-Value
Right arm reach				
MD (% AL)	85.2 ± 12.6	101.3 ± 8.3	<0.001	1.51
IL (% AL)	82.5 ± 13.6	97.9 ± 12.2	<0.001	1.19
SL (% AL)	68.7 ± 15.4	77.3 ± 8.8	0.010	0.68
CS (% AL)	78.8 ± 10.7	92.2 ± 6.3	<0.001	1.52
Left arm reach				
MD (% AL)	88.8 ± 11.7	102.8 ± 7.1	<0.001	1.45
IL (% AL)	85.4 ± 16.0	95.9 ± 11.9	0.006	0.74
SL (% AL)	66.6 ± 14.5	74.2 ± 7.9	0.015	0.77
CS (% AL)	80.3 ± 10.8	91.0 ± 6.5	<0.001	1.20

Data are mean values ± standard deviations. Cohen’s d with 0 ≤ d ≤ 0.49 indicating small, with 0.50 ≤ d ≤ 0.79 indicating moderate, and with d ≥ 0.80 indicating large effects. AL = arm length; BIF = borderline intellectual functioning; CS = composite score; IL = inferolateral; MD = medial; SL = superolateral [13].

## Data Availability

The data generated and analysed during the present study are not publicly available due to ethical restrictions but are available from the corresponding author upon reasonable request.

## Abbreviations

AL: Arm length; AT: anterior; BIF: Borderline intellectual functioning; CS: Composite score; ID: Intellectual disability; IL: Inferolateral; IQ: Intelligence quotient; LL: Leg length; MD: Medial; PL: Posterolateral; PM: Posteromedial; SL: Superolateral; YBT-LQ: Lower Quarter Y Balance Test; YBT-UQ: Upper Quarter Y Balance Test.
